# Li-Decorated *β*_12_-Borophene as Potential Candidates for Hydrogen Storage: A First-Principle Study

**DOI:** 10.3390/ma10121399

**Published:** 2017-12-07

**Authors:** Tingting Liu, Yuhong Chen, Haifeng Wang, Meiling Zhang, Lihua Yuan, Cairong Zhang

**Affiliations:** 1 State Key Laboratory of Advanced Processing and Recycling of No-ferrous Metals, Lanzhou University of Technology, Lanzhou 730050, China; ttlLUT@163.com (T.L.); zhcrxy@lut.cn (C.Z.); 2 School of Science, Lanzhou University of Technology, Lanzhou 730050, China; zhangml_2000@126.com (M.Z.); yuanlh@lut.cn (L.Y.); 3 Department of Physics, College of Science, Shihezi University, Xinjiang 832003, China; whfeng@shzu.edu.cn; 4 The School of Nuclear Science and Technology, Lanzhou University, Lanzhou 730000, China

**Keywords:** *β*_12_-borophene, Li-decorated, hydrogen storage, first-principles calculations

## Abstract

The hydrogen storage properties of pristine *β*_12_-borophene and Li-decorated *β*_12_-borophene are systemically investigated by means of first-principles calculations based on density functional theory. The adsorption sites, adsorption energies, electronic structures, and hydrogen storage performance of pristine *β*_12_-borophene/H_2_ and Li-*β*_12_-borophene/H_2_ systems are discussed in detail. The results show that H_2_ is dissociated into Two H atoms that are then chemisorbed on *β*_12_-borophene via strong covalent bonds. Then, we use Li atom to improve the hydrogen storage performance and modify the hydrogen storage capacity of *β*_12_-borophene. Our numerical calculation shows that Li-*β*_12_-borophene system can adsorb up to 7 H_2_ molecules; while 2Li-*β*_12_-borophene system can adsorb up to 14 H_2_ molecules and the hydrogen storage capacity up to 10.85 wt %.

## 1. Introduction

As the gap between energy supply and demand has become increasingly prominent, sources of renewable energy has been investigated urgently. Hydrogen is an inexhaustible source of clean energy, making it important for society to develop and utilize this energy [1,2]. Hydrogen storage is one of the most critical technical problems in the development of hydrogen energy sources. The average adsorption energy of the ideal physical hydrogen storage method should be between chemical and physical adsorption energy (0.1~0.8 eV) [3,4]. The US Department of Energy (DOE) and the International Energy Agency (IEA) reported that the ideal hydrogen storage capacity should be greater than 5.5 wt % [5]. At present, one of the best types of hydrogen storage methods involves physical adsorption, which results in low adsorption heat, small activation energy, fast hydrogen adsorption and desorption, and reversible cyclization performance. Carbon nanomaterials have become a hotspot of physical hydrogen storage materials due to their characteristics of a large specific surface area, good adsorption kinetic properties and reversible hydrogen storage [6,7]. However, clean carbon nanomaterials adsorb H_2_ molecules with weak binding capacity, which means that they have low hydrogen storage capacity and are not ideal. Therefore, it is essential to find a suitable physical adsorbent.

Recently, 2D (two-dimensional) borophene created from Boron elements was artificially synthesized [8]. Although there are many theoretical studies about the possible 2D borophene structure [9], only three types of stable structures have been synthesized for borophene so far [8,10]. Borophene’s unique metal properties, mechanical properties, and optical properties have been extensively studied [11,12,13,14,15], but only a few studies have considered its hydrogen storage properties. Borophene and graphene [16] have a similar 2D planar structure with a large specific surface area. Moreover, the relative atomic mass of B atom is smaller than the relative atomic mass of C atom. Therefore, we suspect that borophene has better hydrogen storage properties than graphene (it exhibits a triangular lattice with different periodic arrangements and is flat without obvious vertical undulation). Feng et al. [10] reported that *β*_12_-borophene is more stable than the other two types of borophene. Chen et al. [17] used the first-principles method to study the hydrogen storage properties of Ca-*β*_12_-borophene and found that it has a larger adsorption energy compared to other types of borophene. Therefore, we selected *β*_12_-borophene as the research focus. In this work, we performed theoretical calculations for the hydrogen storage properties of pure *β*_12_-borophene and Li-*β*_12_-borophene based on the first-principle study. We found that H_2_ molecules were completely dissociated into two H atoms that were adsorbed on the B–B bridge sites to form H–B covalent bonds, thus making it difficult to dissociate. Comparison of the improvement in hydrogen storage properties of graphene found that the graphene surface was modified by alkali metal (Li, Na, K) [18], alkali-earth metal (Ca) [19], light metal (Al) [20] and transition metals (Cu, Pd, Y) [21,22,23,24], which can change the chemical activity of the graphene surface and could effectively change the hydrogen storagecapability. The quality of alkali metal (Li atoms) is very light, which helps to enhance the hydrogen storage density [25]. The transition metal atom-modified nanostructures are highly reactive and can easily cause the dissociation of H_2_ molecules, which is detrimental to the reversible storage of hydrogen [26]. Therefore, we selected the lightest Li atom to modify the *β*_12_-borophenen. H_2_ adsorbed on Li-*β*_12_-borophene by physical adsorption, which improved the reversible hydrogen storage performance and significantly increased the amount of hydrogen storage. It is expected that this work can provide theoretical support for *β*_12_-borophene being used as hydrogen materials.

## 2. Computational Methods

All density functional theory (DFT) calculations are carried out using the Cambridge Sequential Total Energy Package (CASTEP) [27], and the DFT evaluation is based on the plane-wave expansion. We use the Generalized Gradient Approximation (GGA) with the Perdew-Burke-Ernzerhof (PBE) exchange-correlation functional [28] to describe exchange and correlation effects. The van der waals forces of H_2_ adsorption on Li-*β*_12_-borophenen is modified by DFT-D methods. While the DFT-D perform poorly for energetics in layered materials [29], it is important to deal with the molecules adsorption system. We select the Ultrasoft Pseudopotential [30] to describe the interaction of electron-ion, and the electron wave functions are expanded by plane wave. The convergence tolerance energy, the force on each atoms and displacement convergence criterions are set to 5.0 × 10^−6^ eV/atom, 0.01 eV/Å and 0.001 Å, respectively. All atoms are relaxed in our calculations. In order to eliminate the interaction of the interlayer we select the vacuum thickness 20 Å. Considering the calculation accuracy and computational efficiency, all calculations are using a cutoff energy of 600 eV and 9 × 16 × 5 k-point mesh in the Brillouin zone. 

The adsorption energy ($E_{ads}$) and average adsorption energy (${\bar{E}}_{ads})$ of H_2_ adsorption on Li-*β*_12_-borophene are calculated by the following formulas [31]:

(1) $$E_{ads}=E_{iH2+nLi+\beta 12-\mathrm{borophene}}-E_{\left(i-1\right)H2+n\mathrm{Li}+\beta 12-\mathrm{borophene}}-E_{H2}$$

(2) $$\bar{E_{ads}}=\left(E_{iH2+n\mathrm{Li}+\beta 12-\mathrm{borophene}}-E_{n\mathrm{Li}+\beta 12-\mathrm{borophene}}-iE_{H2}\right)/i$$

The average adsorption energy of Li atom on *β*_12_-borophenec [32] is defined as:

(3) $$\bar{E_{b}}=\left(E_{n\mathrm{Li}+\beta 12-\mathrm{borophene}}-E_{\beta 12-\mathrm{borophene}}-nE_{\mathrm{Li}}\right)/n$$

where $E_{iH2+n\mathrm{Li}+\beta 12-\mathrm{borophene}}$, $E_{\left(i-1\right)H2+\mathrm{nLi}+\beta 12-\mathrm{borophene}}$ and $E_{n\mathrm{Li}+\beta 12-\mathrm{borophene}}$ are the total energy of the *n* Li-*β*_12_-borophene with *i*, *i* − 1 H_2_ molecules and *β*_12_-borophene with *n* Li atoms, respectively. $E_{\beta 12-\mathrm{borophene}}$, $E_{\mathrm{Li}}$ and $E_{H2}$ are the total energy of the *β*_12_-borophene, free Li atom and an isolated H_2_, respectively. *n* is the number of adsorbed Li atoms.

## 3. Results and Discussion

### 3.1. H_2_ Adsorption on β_12_-Borophene

**Figure 1:**
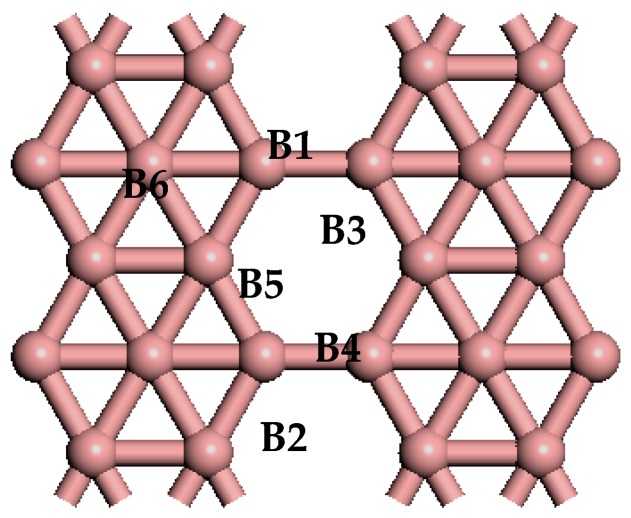
The optimized atomic structure of pure *β*_12_-borophene. The alphanumeric characters on the graph represent the corresponding atoms.

The optimized lattice parameters of the primitive cell of *β*_12_-borophenen are a = 5.069 Å, b = 2.929 Å, agree well with the experimental result (a = 5 Å and b = 3 Å) [10] and other theoretical calculation results [33,34,35]. In our follow calculations, we choose a 2 × 2 unit cell (see Figure 1) of the *β*_12_-borophenen containing 20 B atoms (in Figure 1) to investigate the hydrogen storage adsorbed on *β*_12_-borophene.

**Figure 2:**
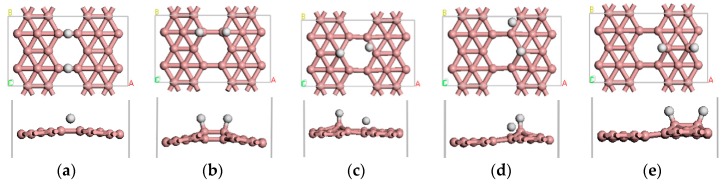
(**a**–**e**) show the five stable optimized geometrical structures of *β*_12_-borophene/H_2._

We first investigated the adsorption behavior of one H_2_ molecule on *β*_12_-borophene. The H_2_ molecule is initially placed in a parallel or vertical direction at different positions of the *β*_12_-borophene plane. We found that there are five stable adsorbed configurations in total, as illustrated in Figure 2. In all cases, the H_2_ molecule is dissociated into two separate H atoms after adsorption and the distance between the H atoms will change from 0.753 to 2.366 Å. Furthermore, the distance between H and its nearest B atom (r_H-B_) greatly increased from 1.217 to 1.358 Å. The most stable case among all the five adsorption configurations is shown in Figure 2a. In this case, the H_2_ molecule dissociated into two H atoms that are adsorbed on the B1–B3 and B2–B4 bridge sites with an $E_{ads}$ value of −0.536 eV, which is related to chemical adsorption. Mulliken analysis demonstrates that there is 0.23 e^−^ transferred from B to H, which occurs mainly in the H 1s orbital and B 2p orbital. The B–H bond population is 0.4, indicating it is a covalent bond, with difficult desorption of the *β*_12_-borophene/H_2_ system. In addition, we further studied the transition states of the stable adsorption configurations by combining linear synchronous and quadratic synchronous transits [21,36]. We found that the most stable adsorption configurations of the activation energy barrier from the reactant to transition state was 1.584 eV, which is smaller than the activation energy barrier of other adsorption methods, indicating it was difficult for the reaction of the H_2_ molecules adsorbed on the surface to take place. 

### 3.2. H_2_ Adsorption on Li-β_12_-Borophene

#### 3.2.1. The Adsorption Structure of Li-*β*_12_-Borophene

It is well known that doping alkali metal atoms to modify hydrogen storage materials may can greatly improve the hydrogen storage properties and increase the hydrogen storage capacity. Specially, lithium (Li) has been widely employed to functionalize 2D materials and improve the hydrogen storage ability. Therefore, in the following section, we chose to add Li atoms to modify the hydrogen storage properties of *β*_12_-borophene.

**Figure 3:**
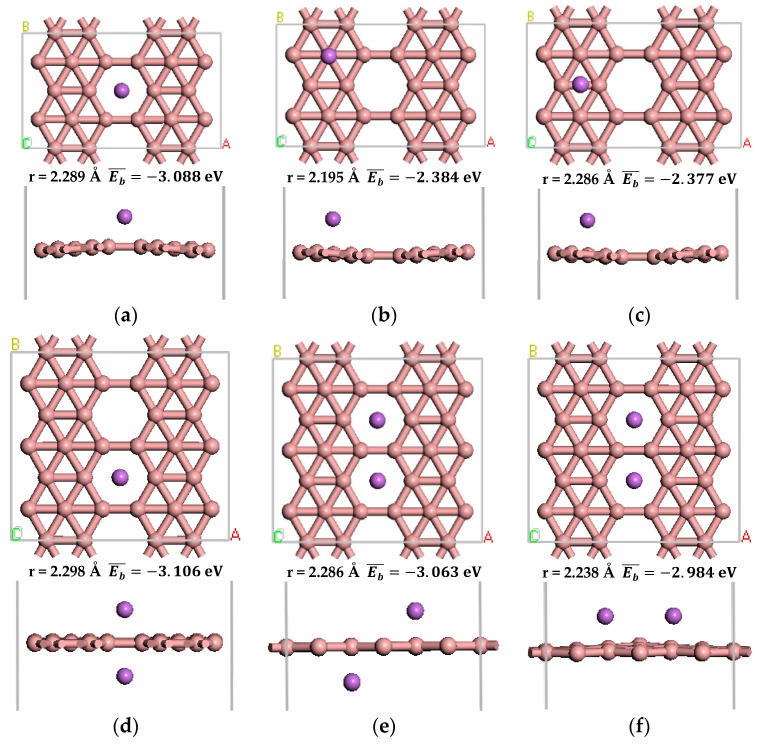
The optimized atomic structure of Li atom decorated *β*_12_-borophene. (**a**–**c**) show the one Li atom decorated single-sided *β*_12_-borophene, respectively. (**d**–**f**) show the two Li atoms decorated double-sided *β*_12_-borophene, respectively.

We examined the adsorption of Li atoms on pure *β*_12_-borophene. After optimization, we obtained three different stable adsorption structures, as shown in Figure 3a–c. Similar to Li-decorating graphene [37], the most favorable Li adsorption site on *β*_12_-borophene is the hollow center of B ring (Figure 3a).

Doping alkali metal atoms to modify hydrogen storage materials requires the average adsorption energy of the metal atoms on the substrate to be greater than the cohesive energy of the metal atoms in the solid form [38]. The average adsorption energy of Li atom on the *β*_12_-borophene is −3.088 eV, which is significantly greater than the cohesive energy of −1.795 eV of Li [39]. This indicates that Li atoms can be dispersed uniformly on *β*_12_-borophene, instead of forming metal clusters.

There are three stable adsorption structures of two Li atoms after adsorption on the *β*_12_-borophene as shown in Figure 3d–f, respectively. One of the most stable adsorption sites involves the two Li atoms being located on both sides of the same B ring. The distance between Li and the nearest B is 2.298 Å. The average adsorption energy is −3.106 eV, which is larger than the cohesive energy of Li atoms. After optimization, the relaxation of *β*_12_-borophene is very small. Each Li atom in the Li-*β*_12_-borophene system is an active adsorption site, allowing a large number of H_2_ molecules to be adsorbed around the Li atom in order to significantly increase the hydrogen storage capacity.

**Figure 4:**
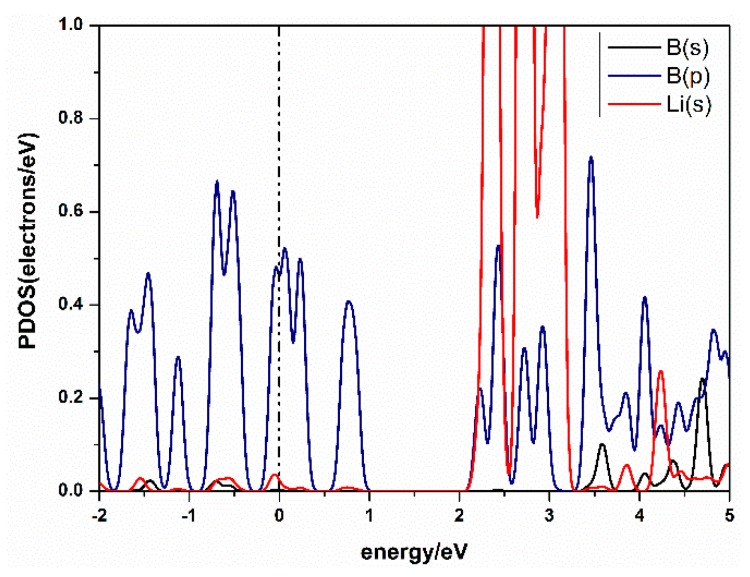
Partial density of states (PDOS) of Li-decorated *β*_12_-borophene system.

The charge transfer between atoms can be analyzed by Mulliken analysis [40], which shows that the charge was transferred from Li to B. From the Partial Densities of States (PDOS) of the Li-*β*_12_-borophene structure in Figure 4, we found the peak of B atom’s 2p orbital overlaps with the peak of the Li atom’s 1s orbital. This suggests a strong hybridization between B and Li atoms. A similar binding mechanism has also been confirmed in other metal-modified nanostructures [41]. In addition, it can be seen from the PDOS that the metal properties of *β*_12_-borophene did not change after modification of Li atom.

#### 3.2.2. Adsorption of H_2_ Molecules on Li-*β*_12_-Borophene

**Figure 5:**
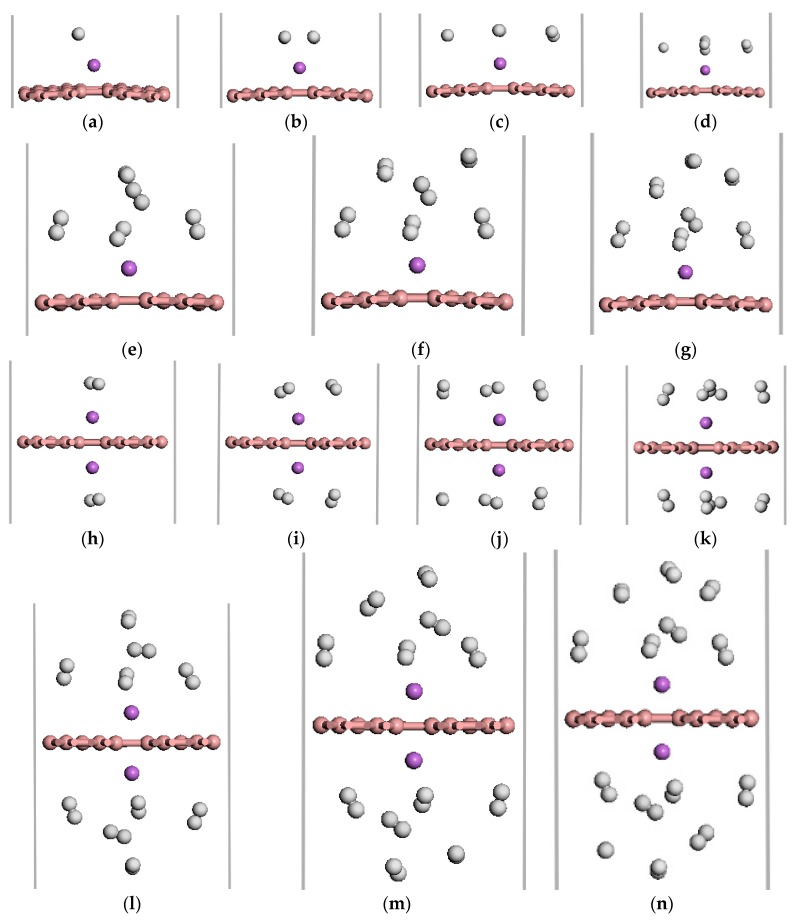
The optimized atomic structures of the Li-*β*_12_-borophene/H_2_. (**a**–**g**) are 1~7 H_2_ molecules adsorption on Li-*β*_12_-borophene system. (**h**–**n**) are 2~14 H_2_ molecules adsorption on 2Li-*β*_12_-borophene system. The pink, purple and white balls in this and aforementioned figures express B, Li and H atoms, respectively.

We investigated the adsorption properties of H_2_ molecules on Li-*β*_12_-borophene. Figure 5 shows the optimized geometries of 1–7 H_2_ molecules adsorbed on the Li-modified *β*_12_-borophene. Table 1 lists the adsorption energy and average adsorption energy calculated by the GGA PBE functional and DFT-D methods. First, we investigate the adsorption sites of H_2_ molecules on Li-*β*_12_-borophene. For the first adsorbed H_2_ molecules, many adsorption sites were considered in order to find the most stable site. The most stable site involves H_2_ being parallel to the *β*_12_-borophene plane, which is opposite to the H_2_ vertical adsorption on Ca-*β*_12_-borophene [17]. After adsorption, the corresponding r_H-H_ of the adsorbed H_2_ is 0.756 Å, which is larger than the distance of free H_2_ (0.753 Å). To investigate the maximum storage capacity of single Li atom-modified *β*_12_-borophene, more H_2_ was added around Li gradually. The minimum distance between the H and Li atom are range of 2.164 to 6.368 Å. The first four H_2_ molecules were parallel to the *β*_12_-borophene and were around the Li atom at the same level. When the fifth H_2_ molecule was added to the system, two H_2_ molecules moved to an upper layer after relaxation. This may be due to the limited space around the Li atom and the repulsive interactions between the adsorbed H_2_. The average adsorption energy slowly reduced from −0.385 to −0.210 eV/H_2_ due to the strong steric interactions between the adsorbed H_2_. Interestingly, the adsorption energy suddenly rose to –0.388 eV after the second H_2_ molecule was added to the system. With an increase in the number of H_2_ molecules, the H_2_ molecules becomes further away from the Li atom and the adsorption weakens. The average adsorption energy was at its minimum (−0.210 eV/H_2_) when the seventh H_2_ molecule was adsorbed. At this time, the hydrogen storage capacity reached 5.90 wt %, which exceeded the ideal hydrogen storage capacity (over 5.5 wt %). In order to further increase the hydrogen storage capacity, we added two Li atoms to decorate the *β*_12_-borophene to adsorb H_2_ molecules. 2Li-*β*_12_-borophene can adsorb up to 14 H_2_ molecules and the minimum average adsorption energy is −0.220 eV. The hydrogen storage capacity can reach up to 10.85 wt %, which is larger than the hydrogen storage capacity with a gravimetric hydrogen density of 9.5 wt % of the Ca-*β*_12_-borophene/H_2_ system [17]. The optimized structure is shown in Figure 5h–n. The average adsorption energy (${\bar{E}}_{ads}$) is in the range of −0.381 to −0.220 eV/H_2_, which is necessary for practical application [3,4]. In addition, the calculated adsorption energy and average energy of H_2_ adsorption by DFT-D method are larger than those calculated by the GGA PBE functional, but the overall adsorption method has not changed.

#### 3.2.3. Electronic Properties of Li-*β*_12_-Borophene/H_2_

**Figure 6:**
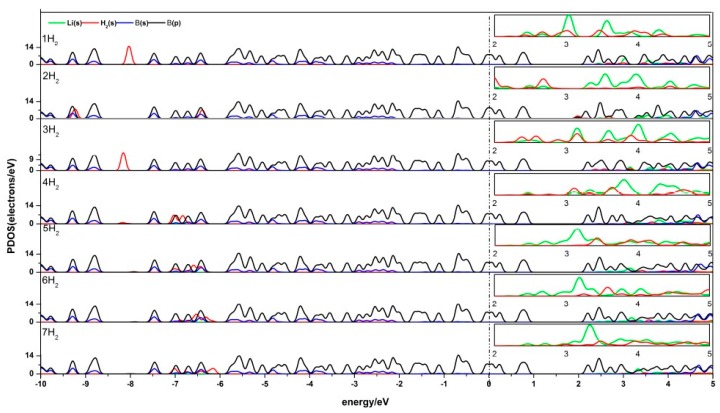
PDOS of Li-*β*_12_-borophene with 1–7 H_2_ molecules adsorbing. (The PDOS of Li-s orbit and H-s orbit in the range of 2.0 eV~5.0 eV is enlarged as shown in the small box above each corresponding figure.)

The density of states (DOS) reflects the number of states of the unit energy, which is important in further understanding the interaction between H_2_ and Li-*β*_12_-borophene. The partial density of states (PDOS) of Li-*β*_12_-borophene/H_2_ is shown in Figure 6. Obvious hybridizations between the Li-s orbit and H-s orbit can be found in 2.0 eV~5.0 eV, which demonstrates a strong interaction between H_2_ and Li atoms. With an increase in the number of H_2_ molecules, the peak values of H_2_ molecules become smaller and further away from the fermi level. This indicates that the interaction between H_2_ molecules and Li-*β*_12_-borophene weakens, which is consistent with the average adsorption energy becoming smaller. Another overlap between the B-p and H-s orbits was found at –10.0 eV~5.0 eV. Upon the adsorption of the second H_2_ molecule, the H 1s orbit peaks move to the lift, implying an increased stability in the system. This is consistent with the increase in the Eads value after the second H_2_ addition. With an increase in the number of H_2_ molecules (an expected in the second H_2_), the H-s orbits move to the right and the peak values become smaller, which indicates that the interaction between H_2_ and *β*_12_-borophene becomes increasingly weaker. This conclusion is consistent with the decrease in the average adsorption energy $({\bar{E}}_{ads}$). The B-p and Li-s orbits also have hybridization, which implies an interaction between the Li and B atoms. The peaks near and below the fermi surface are mostly contributed by the B-s orbits, which means that the H_2_ molecules and Li atom have less influence on the *β*_12_-borophene. The comparison of the PDOS of a single Li-*β*_12_-borophene show that the interaction between Li atom and *β*_12_-borophene is weakened due to the adsorption of H_2_ molecules. The PDOS of two Li-*β*_12_-borophene/H_2_ consistent with this analysis.

The bonding strength between atoms can be quantitatively analyzed based on the Mulliken charge population and bond population. Table 2 shows the Mulliken charge population before and after one H_2_ moleculebecomes absorbed on the Li-*β*_12_-borophene. H (1) and H (2) represent the two H atoms of the adsorbed H_2_ molecule; while B1, B5 and B6 are three B atoms that transfer the greatest amount of charge in the *β*_12_-borophene (as shown in Figure 1). The two H atoms have charges of 0.06 e and 0.05 e, respectively. In contrast, the Li atom loses 1.40 e, which occurs mainly in the H and Li atomic orbits. The Li atom transfers charge to the H_2_ molecules, resulting in the H_2_ molecules carrying more negative charge and Li atom showing positive charge. The interaction between the H_2_ molecules and the Li atom is consistent with the conclusion of the PDOS analysis. In addition, the B atoms obtains charge, with this charge transfer mainly occurring in the B-2p orbits and H-s orbits. This is in contrast with the Mulliken charge population of the *β*_12_-borophene/H_2_, in which the charge transfer mainly occurs in H and B atoms forming a covalent bond of H–B that is not favorable for the desorption of H_2_. Due to the *β*_12_-borophene being modified by Li atoms, H_2_ molecules and B atoms only have small interactions, resulting in the H_2_ molecules physically adsorbing on the Li-*β*_12_-borophene. This is conducive for H_2_ desorption and increases the hydrogen storage capacity.

## 4. Conclusions

In summary, we performed a study on hydrogen storage properties of pure *β*_12_-borophene and Li-decorated *β*_12_-borophene through DFT calculations. It is found that H_2_ molecules are mainly adsorbed on pure *β*_12_-borophene as chemical adsorption with an adsorption energy of −0.536 eV. The H_2_ molecules are dissociated into two H atoms, which tend to the bridge of two B site and the H–B bond to form covalent bond. In order to improve the hydrogen storage performance of pure *β*_12_-borophene and increase the hydrogen storage capacity, we use the Li atom to modify the *β*_12_-borophene. It is found that a single Li atom adsorbed on the center of Boron ring with the adsorption energy −3.088 eV, the Li-*β*_12_-borophene can adsorb up to 7 H_2_ molecules with the average adsorption energy of −0.210 eV/H_2_. The charge transfer of the Li-*β*_12_-borophene/H_2_ is that H and B atoms lose electron, Li atom get electron. We use two Li atoms to modify *β*_12_-borophene to increase its hydrogen storage capacity. It is find that the two Li atoms are located at the same position on both sides of the same boron hole. 2Li-*β*_12_-borophene system can adsorb up to 14 H_2_ molecules and the hydrogen storage capacity up to 10.85 wt %. The average adsorption energy is range of −0.381 to −0.220 eV/H_2_, which is necessary for practical application [3,4].

## Figures and Tables

**Table 1:** The adsorption energy, average adsorption energy, the distance between H and H (r_H-H_), the distance between H and Li of Li-*β*_12_-borophene system (r_H-Li_).

Li-*β*_12_-borophene	**Number of H_2_**	**1 H_2_**	**2 H_2_**	**3 H_2_**	**4 H_2_**	**5 H_2_**	**6 H_2_**	**7 H_2_**
	$E_{ads}$/eV	−0.247	−0.281	−0.154	−0.179	−0.139	−0.169	−0.134
	$E_{ads}$ (DFT-D)/eV	−0.385	−0.388	−0.251	−0.147	−0.160	−0.167	−0.142
	${\bar{E}}_{ads}$/eV/H_2_	−0.247	−0.213	−0.194	−0.190	−0.181	−0.178	−0.173
	${\bar{E}}_{ads}$ (DFT-D)/eV/H_2_	−0.385	−0.387	−0.286	−0.251	−0.233	−0.222	−0.210
	r_H-H/_Å	0.756	0.757	0.753	0.755	0.753	0.753	0.753
	r_H-Li/_Å	2.164	2.169	3.813	3.810	4.661	5.667	6.368
2Li-*β*_12_-borophene	**Number of H_2_**	**2 H_2_**	**4 H_2_**	**6 H_2_**	**8 H_2_**	**10 H_2_**	**12 H_2_**	**14 H_2_**
	${\bar{E}}_{ads}$ (DFT-D)/eV/H_2_	−0.381	−0.298	−0.274	−0.262	−0.230	−0.226	−0.220

**Table 2:** Mulliken population analysis of the Li-*β*_12_-borophene before and after one H_2_ molecule adsorption.

Atom	Mulliken					
	Before Adsorption/e			After Adsorption/e		
	s	p	Charge	s	p	Charge
H (1)	1.0			1.06		−0.06
H (2)	1.0			1.05		−0.05
B1	0.82	2.18	0	0.83	2.36	−0.19
B5	0.74	2.23	0.03	0.75	2.40	−0.15
B6	0.65	2.40	−0.05	0.65	2.40	−0.05
Li	3	0	0	1.60		1.40

## References

[1] Song Y., Guo Z.X., Yang R. (2004). Influence of selected alloying elements on the stability of magnesium dihydride storage applications: A first-principles investigation. Phys. Rev. B.

[2] Schlapbach L., Züttel A. (2001). Hydrogen-storage materials for mobile applications. Nature.

[3] Rosi N.L., Eckert J., Eddaoudi M., Vodak D.T., Kim J., O’Keeffe M., Yaghi O.M. (2003). Hydrogen storage in microporous metal-organic frameworks. Science.

[4] Han S.S., Goddard W.A. (2007). Lighium-doped metal-organic frameworks for reversible H_2_ storage at ambient temperature. J. Am. Chem. Soc..

[5] U.S. Department of Energy (2014). Hydrogen, Fuel Cells Program: FY Annual Progress Report.

[6] Seenithurai S., Pandyan R.K., Kumar S.V., Saranya C., Mahendran M. (2014). Al-decorated carbon nanotube as the molecular hydrogen storage medium. Int. J. Hydrog. Energy.

[7] Wu M.H., Gao Y., Zhang Z.Y., Zeng X.C. (2012). Edge-decorated graphene nanoribbons by scandium as hydrogen storage media. Nanoscale.

[8] Mannix A.J., Zhou X.F., Kiraly B., Wood J.D., Alducin D., Myers B.D., Liu X., Fisher B.L., Santiago U., Guest J.R. (2015). Synthesis of borophenes: Anisotropic, Two-dimensional boron polymorphs. Science.

[9] Zhang Z.H., Yang Y., Gao G.Y., Yakobson B.I. (2015). Two-Dimensional Boron Monolayers Mediated by metal Substrates. Angew. Chem. Int. Ed. Engl..

[10] Feng B.J., Zhang J., Zhong Q., Li W.B., Li S., Li H., Cheng P., Meng S., Chen L., Wu K.H. (2016). Experimental realization of two-dimensional boron sheets. Nat. Chem..

[11] Feng B.J., Zhang J., Liu R.Y., Iimori T., Lian C., Li H., Chen L., Wu K.H., Meng S., Komori F. (2016). Direct evidence of metallic bands in a monolayer boron sheet. Phys. Rev. B.

[12] Padilha J.E., Miwa R.H., Fazzio A. (2016). Directional dependence of the electronic and transport properties of 2D borophene and borophane. Phys. Chem. Chem. Phys..

[13] Mortazavi B., Rahaman O., Dianat A., Rabczuk T. (2016). Mechanical responses of borophene sheets: A first-principles study. Phys. Chem. Chem. Phys..

[14] Wang H.F., Li Q.F., Gao Y., Miao F., Zhou X.F., Wan X.G. (2016). Strain effects on borophene: Ideal strength, negative Possion’s ration and phonon instability. New J. Phys..

[15] Liu Y., Dong Y.J., Tang Z., Wang X.F., Wang L., Hou T.G., Lin H.P., Li Y.Y. (2016). Stable and metallic borophene nanoribbons from first-principles calculations. J. Mater. Chem. C.

[16] Novoselov K.S., Geim A.K., Morozov S.V., Jiang D., Zhang Y., Dubonos S.V., Grigorieva I.V., Firsov A.A. (2004). Electric field effect in atomically thin carbon films. Science.

[17] Chen X.F., Wang L., Zhang W.T., Zhang J.L., Yuan Y.Q. (2017). Ca-decorated borophene as potential candidates for hydrogen storage: A first-principle study. Int. J. Hydrog. Energy.

[18] Wang Y.H., Meng Z.S., Liu Y.Z., You D., Wu K., Lv J., Wang X.Z., Deng K.M., Rao D., Lu R.F. (2015). Lithium decoration of three dimensional boron-doped graphene frameworks for high-capacity storage. Appl. Phys. Lett..

[19] Reunchan P., Jhi S.H. (2011). Metal-dispersed porous graphene for hydrogen storage. Appl. Phys. Lett..

[20] Ao Z.M., Jiang Q., Zhang R.Q., Tan T.T., Li S. (2009). Al doped graphene: A promising material for hydrogen storage at room temperature. J. Appl. Phys..

[21] Faye O., Eduok U., Szpunar J., Szpunar B., Samoura A., Beye A. (2017). Hydrogen Storage on bare Cu atom and Cu-functionalized boron-doped graphene: A first principles study. Int. J. Hydrog. Energy.

[22] Faye O., Szpunar J.A., Szpunar B., Beye A.C. (2017). Hydrogen adsorption and storage on Palladium-functionalized graphene with NH-dopant: A first principles calculation. Appl. Surf. Sci..

[23] Yuan L.H., Chen Y.H., Kang L., Zhang C.R., Wang D.B., Wang C.N., Zhang M.L., Wu X.J. (2017). First-principles investigation of hydrogen storage capacity of Y-decorated porous graphene. Appl. Surf. Sci..

[24] Zhao Y., Kim Y.H., Dillion A.C., Heben M.J., Zhang S.B. (2005). Hydrogen storage in novel organometallic buckyballs. Phys. Rev. Lett..

[25] Yoon M., Yang S.Y., Hicke C., Wang E., Geohegan D., Zhang Z.Y. (2008). Calcium as the superior coating metal in functionalization of carbon fullerenes for high-capacity hydrogen storage. Phys. Rev. Lett..

[26] Guo J., Liu Z.G., Liu S.Q., Zhao X.H., Huang K.L. (2011). High-capacity hydrogen storage medium: Ti doped fullerene. Appl. Phys. Lett..

[27] Clark S.J., Segall M.D., Pickard C.J., Hasnip P.J., Probert M., Refson K.R., Payne M.C. (2005). First Principles methods using CASTEP. Z. Kristallogr..

[28] Perdew J.P., Burke K., Ernzerhof M. (1996). Generalized Gradient Approximation Made Simple. Phys. Rev. Lett..

[29] Bjorkman T., Gulans A., Krasheninnikov A.V., Nieminen R.M. (2012). Van der Waals Bonding in Layered Compounds from Advanced Density-Functional First-Principles Calculations. Phys. Rev. Lett..

[30] Vanderbilt D. (1990). Soft self-consistent pseudopotentials in generalized eigenvalue formalism. Phys. Rev. B.

[31] Hu W., Xia N., Wu X., Li Z., Yang J. (2014). Silicene as a highly sensitive molecule sensor for NH_3_, NO and NO_2_. Phys. Chem. Chem. Phys..

[32] Chen Y.H., Wang J., Yuan L.H., Zhang M.L., Zhang C.R. (2017). Sc-Decorated Porous Graphene for High-Capacity Hydrogen Storage: First-Principles Calculations. Materials.

[33] Wu X., Dai J., Zhuo Z., Yang J., Zeng X. (2012). Two-Dimensional Boron Monolayer Sheets. ACS Nano.

[34] Peng B., Zhang H., Shao H., Ning Z., Xu Y., Ni G., Lu H., Zhang D., Zhu H. (2017). Stability and strength of atomically thin borophene from first principles calculations. Mater. Res. Lett..

[35] Peng B., Zhang H., Shao H.Z., Xu Y.F., Zhang R.J., Zhua H.Y. (2016). Electronic, Optical, and thermodynamic properties of borophene from first-principle calculations. J. Mater. Chem. C.

[36] Pan C.C., Chen Y.H., Wu N., Zhang M.L., Yuan L.H., Zhang C.R. (2017). A First Principles Study of H_2_ Adsorption on LaNiO_3_(001) Surfaces. Materials.

[37] Ataca C., Akturk E., Ciraci S., Ustunel H. (2008). High-capacity hydrogen storage by metallized graphene. Appl. Phys. Lett..

[38] Sun Q., Wang Q., Jena P., Kawazoe Y. (2005). Clustering of Ti on a C60 surface and its effect on hydrogen storage. J. Am. Chem. Soc..

[39] Doll K., Harrison N.M., Saunders V.R. (1999). A density functional study of lithium bulk and surfaces. J. Phys.-Condens. Matter.

[40] Mulliken R.S. (1955). Molecular Compounds and Their Spectra. V. Orientation in Molecular Complexes. J. Chem. Phys..

[41] An H., Liu C.S., Zeng Z., Fan C., Ju X. (2011). Li-doped B2C graphene as potential hydrogen storage medium. Appl. Phys. Lett..

